# HIERARCHICAL ANALYSIS OF THE FACTORS ASSOCIATED WITH ANEMIA IN INFANTS

**DOI:** 10.1590/1984-0462/;2018;36;3;00013

**Published:** 2018

**Authors:** Elma Izze da Silva Magalhães, Daniela Santana Maia, Michele Pereira, Joel Alves Lamounier, Daniela da Silva Rocha

**Affiliations:** a Universidade Federal de Pelotas, Pelotas, RS, Brasil.; b Universidade Estadual do Sudoeste da Bahia, Vitória da Conquista, BA, Brasil.; c Universidade Federal de Juiz de Fora, Juiz de Fora, MG, Brasil.; d Universidade Federal de São João del-Rei, Divinópolis, MG, Brasil.; e Universidade Federal da Bahia, Vitória da Conquista, BA, Brasil.

**Keywords:** Anemia, Infant, Social class, Iron, dietary, Risk factors, Anemia, Lactente, Nível socioeconômico, Ferro na dieta, Fatores de risco

## Abstract

**Objective:**

To evaluate the prevalence of anemia and the associated factors in infants assisted in health units of Vitória da Conquista, Bahia, Northeast Brazil.

**Methods:**

Cross-sectional study with a representative sample of 366 children aged 6 to 23 months. A questionnaire was applied to the caregiver, and the children’s anthropometric measurements and hemoglobin levels were collected. The associations were identified by Poisson regression with robust variances based on a hierarchical analysis model.

**Results:**

The prevalence of anemia was 26.8%, and the associated factors were: family income equal to or lower than one minimum wage (PR: 1.50; 95%CI 1.03-2.18), number of household members higher than five (PR: 1.50; 95%CI 1.07-2.11), use of unfiltered water (PR: 1.68; 95%CI 1.11-2.56), number of offspring higher than three (PR: 1.64; 95%CI 1.01-2.68), consumption of meat and/or viscera less than once/week (PR: 1.78; 95%CI 1.24-2.58) and age 6-11 months (PR: 1.75; 95%CI 1.20-2.55).

**Conclusions:**

Anemia in the infants assessed is a moderate public health problem, which is associated with socioeconomic, demographic, and dietary factors; thus, measures are necessary for its prevention.

## RESUMO

**Objetivo:**

Avaliar a prevalência e os fatores associados à anemia em lactentes assistidos por Unidades de Saúde de Vitória da Conquista, Bahia.

**Métodos:**

Estudo transversal com uma amostra representativa de 366 crianças de 6 a 23 meses. Realizou-se aplicação de questionário ao cuidador, avaliação antropométrica e dosagem de hemoglobina das crianças. As associações foram identificadas por meio da regressão de Poisson com variâncias robustas com seleção hierárquica das variáveis independentes.

**Resultados:**

A prevalência de anemia foi de 26,8%, e os fatores associados foram: renda familiar igual ou inferior a um salário mínimo (RP: 1,50; IC95% 1,03-2,18), número de moradores no domicílio superior a cinco (RP: 1,50; IC95% 1,07-2,11), utilização de água não filtrada (RP: 1,68; IC95% 1,11 -2,56), número de filhos maior que três (RP: 1,64; IC95% 1,01-2,68), consumo de carne e/ou vísceras menor que uma vez por semana (RP: 1,78; IC95% 1,24-2,58) e idade de 6 a 11 meses (RP: 1,75; IC95% 1,20-2,55).

**Conclusões:**

A anemia nos lactentes avaliados é um moderado problema de saúde pública, a qual está associada a fatores socioeconômicos, demográficos e dietéticos. Dessa forma, medidas são necessárias para sua prevenção.

## INTRODUCTION

Anemia can be defined as an abnormal reduction in hemoglobin concentration in the blood as a result of the lack of one or more essential nutrients, with iron deficiency being one of its most important causes, contributing to approximately 90% of the existing anemia types.^1^

It is estimated that a quarter of the world’s population is affected by anemia, which makes it a global public health problem.^2^ In Brazil, data from the National Demographic and Child Health Survey (PNDS) found a prevalence of anemia of 20.9% in children aged under 5 years. This difference was found in a review study in which the prevalence of anemia in children receiving care in health services ranged from 55.1 to 89.1%, with a weighted average of 60.2%.^3^

Several factors contribute to the decrease in hemoglobin concentration and the increase in prevalence in children aged under 2 years. Among these biological, socioeconomic, environmental, health, and nutrition factors, the following can be highlighted: age under 12 months, low birth weight, low socioeconomic level, early weaning, early introduction of cow milk, insufficient iron intake, and even the presence of intestinal parasites.^4^ ^,^ ^5^

In childhood, iron-deficiency anemia is associated with impairments in mental and psychomotor development, early childhood growth retardation, and increase of the morbidity and mortality risk, reducing resistance to infections.^6^ Therefore, prevention interventions to approach the problem have been proposed at a population level, with good responses in the reduction and control of anemia, using different methods.^7^ ^,^ ^8^ ^,^ ^9^ However, the prevalence rates of anemia in Brazilian children are still high.^10^

Thus, considering the great impact of anemia on children’s health, this study aimed to evaluate the prevalence of anemia and the associated factors in infants assisted in Health Units of a municipality in the southwestern region of Bahia.

## METHOD

This is a cross-sectional study, carried out from May 2010 to June 2011, with a sample of children aged 6-23 months, attended at the 21 Health Units of the urban area of the municipality of Vitória da Conquista, located in the southwestern region of Bahia.

Vitória da Conquista is the third largest municipality in the State of Bahia, and its economy is focused mainly on the services sector. According to data from the Brazilian Institute of Geography and Statistics (IBGE), in 2010, the municipality had a Human Development Index (HDI) of 0.678 and a population of 306,866 inhabitants.^11^ The basic care network is composed of 21 Health Units, with 15 Family Health Units, 3 Primary Care Polyclinics, and 3 Health Centers.

The sample calculation was performed using the StatCalc tool from the Epi Info 6.04 software (Centers for Disease Control and Prevention, Atlanta, United States of America), considering the total number of children, aged 6-23 months, receiving care at Urban Health Units in the municipality of Vitória da Conquista (n=6,764); a prevalence of anemia estimated at 25.5%;^12^ accuracy of 5%; confidence level of 95%; with an increase of 20% for possible losses and 10% for multivariable analysis. This calculation resulted in a minimum sample size of 360 children.

The total number of individuals evaluated in each health institution was determined by means of proportional sampling, considering the weight of each health unit in relation to the total number of children, aged between 6 and 23 months, attended in the 21 Health Units of the urban zone of Vitória da Conquista. Subsequently, the children evaluated were randomly selected from a list of children aged 6 to 23 months, who were receiving care at each health unit.

Data were collected by students of the Nutrition course, previously trained to apply the questionnaire, to collect blood by digital puncture and to perform anthropometric measurements. Data collection was carried out through prior appointment in health institutions, on the days of growth and development (GD) consultations. The parents or guardians of the children drawn, who were at the waiting room for the GD consultation, were invited to participate, received clarification on the study objectives, and were requested to sign an Informed Consent upon agreeing to the child’s participation in the study. Data collection was carried out two to three times in each unit to try to include the children drawn. In addition, the health agents and the nursing professionals made appointments for the children drawn and handed them a slip informing the day of the consultation. Despite this, there were 28 guardians who did not attend, and 6 who did not agree to participate in the study. To replace them, a new draw was made.

The following inclusion criteria were adopted: being older than 6 and younger than 24 months and absence of chronic diseases or conditions that could interfere with the child’s health status. Children that were accompanied by guardians who were not able to respond to the collection instrument were excluded - as in the case of individuals who did not live with the child on a daily basis, and thus did not know how to respond accurately to the study questions -, as well as in cases where the child’s companion was a minor.

A Hemocue Hb201^®^ portable hemoglobinometer (Biodina, Rio de Janeiro, Brazil) was used for capillary blood sampling to determine the serum hemoglobin concentration. The cutoff points used to diagnose anemia and severe anemia were 11.0 and 9.5 g/dL, respectively.^13^

The anthropometric evaluation was performed according to the techniques established by the Food and Nutrition Surveillance System (SISVAN) and recommended by the Ministry of Health.^14^ The weight was verified in portable digital electronic scale MarteLC200PP^®^ (Marte Científica, São Paulo, Brazil), with a bearing capacity of up to 200 kg and sensitivity of 50 g, and a tray-type plate (in ABS plastic) was attached for the child’s placement. Length was measured with an Alturexata^®^ (Alturexata, Belo Horizonte, Brazil), with an extension of 0.35 to 2.13 m and an accuracy of 1 mm. The nutritional status of the children was evaluated according to the critical values in the Z scores of the anthropometric indices recommended by the World Health Organization (WHO),^15^ with the help of WHO Anthro Plus^®^ software (World Health Organization, Geneva, Switzerland).

Information on demographic and socioeconomic conditions, as well as maternal and health characteristics and the child’s breastfeeding, feeding, and supplementation practices were obtained through a structured questionnaire applied in the form of interviews with parents or guardians.

The independent variables were categorized based on cut-off points of published articles:

- Socioeconomic: paternal schooling (<8 years of schooling/≥8 years of schooling), maternal schooling (<8 years of schooling/≥8 years of schooling), paternal work (yes/no), maternal work (yes/no), household income (≤1 minimum wage/>1 minimum wage), number of residents in the household (≤5 residents/>5 residents), sewage network (yes/no), sanitary installation (yes/no), garbage collection (yes/no), filtered water (yes/no).
- Maternal: maternal age (<20 years/≥20 years), number of children (≤3 children/>3 children), number of children aged under 5 years (<2 children/≥2 children), type of delivery (normal/cesarean).
- Raw materials for breastfeeding, feeding and supplementation with iron: single breastfeeding (yes/no); exclusive breastfeeding (yes/no); time of exclusive breastfeeding (6 months/6 months or >6 months), consumption of meat and/or viscera (<1 time per week/≥1 times per week), bean consumption (daily/non-daily), dark green leafy vegetable consumption (daily/non-daily), use of iron supplement (yes/no).
- Relating to the child: gender (female/male), age (6 to 11 months/12 to 23 months), skin color (white/non-white), gestational age at birth (<37 weeks/≥37 weeks), weight at birth (<2,500 g/≥2,500 g), previous hospitalization (yes/no), weight/age index (≥2 Z score/<2 Z score), height/age index (≥2 Z score/<2 Z score), weight/height index (≥2 Z score/<2 Z score).

**Figure 1:**
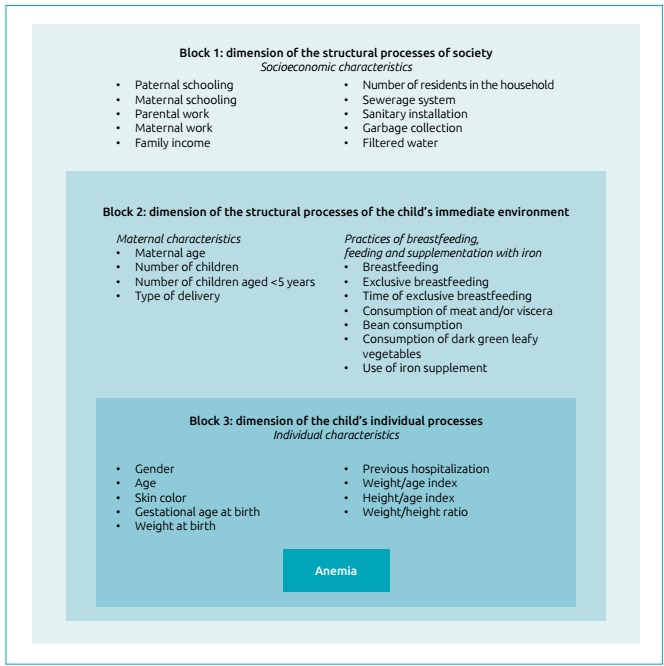
Hierarchical conceptual model of the determinants of childhood anemia, adapted from Silva, Giugliani and Aerts^21^.

Statistical analyzes were performed in the Stata software version 12 (StataCorp, College Station, Texas, United States of America). The normality of distribution of the continuous variables was evaluated using histograms and the Shapiro-Wilk test. To characterize the study population, the categorical variables were described by means of absolute and relative frequencies, and the quantitative variables were described through central tendency and dispersion measures. In order to verify the factors associated with anemia in children, a bivariate analysis was performed initially with estimates of crude prevalence ratios and respective confidence intervals. Then, the Poisson regression was used with robust variances, and the variables that presented statistical significance at a level of 20% (p<0.20) were selected for inclusion in the multivariate model. In the multivariate analysis, the hierarchical input of the variables was performed in blocks, in the following order:

- Block 1: socioeconomic variables.
- Block 2: maternal variables and practices of breastfeeding, feeding and supplementation with iron.
- Block 3: individual variables of the child, according to a conceptual model to determine childhood anemia (Figure 1), adapted from the model proposed by Silva, Giugliani and Aerts.^4^

The variables of the most distal blocks remained as adjustment factors for those of the hierarchically inferior blocks. The statistically significant association (p<0.05) between a given study factor and anemia, after adjusting for the potential factors of the same block and upper hierarchical blocks, indicates the existence of an independent effect of this factor. The quality of the adjustment of the regression model was evaluated by the Akaike criterion (AIC), by comparing the values obtained in each model to select the best model, characterized as the one in which the lowest score was obtained in this criterion.

The research project was submitted and approved by the Research Ethics Committee of Universidade Estadual da Bahia (CEP/UESB), under protocol no. 048/2010, and the study was conducted in accordance with the ethical standards established in the Declaration of Helsinki of 1964, and its subsequent amendments. Children diagnosed with anemia were referred to the health services of the municipality for treatment with iron salts and follow-up by health professionals.

## RESULTS

Of the 366 children evaluated, 26.8% had anemia, of which 20.4% had severe anemia. Half of the studied population was male, and 45.6% were aged between 6 and 11 months, with an average of 13.9 (standard deviation 5.2) months of age. The monthly income of 82.6% of the families was equal to or less than a minimum wage, valid at the time of the study, and the median income was BRL 510.00 (Table 1).

**Table 1:** Crude prevalence ratios of anemia and its 95% confidence intervals, according to the socioeconomic characteristics of children aged 6 to 23 months attended by Health Units of Vitória da Conquista, Bahia, Brazil, 2010/2011 (n=366).

	n (%)	Prevalence of anemia (%)	PR (crude)	95%CI	p-value^a^
Family income (minimum wage)					
>1	61 (17.4)	23.9	1	1.19-2.47	0.004*
≤1	289 (82.6)	41.0	1.72		
Paternal schooling (years of study)					
≥8	175 (53)	22.3	1	1.03-2.11	0.032*
<8	155 (47)	32.9	1.48		
Maternal schooling (years of study)					
≥8	227 (62.7)	22.5	1	1.08-2.13	0.016*
<8	135 (37.3)	34.1	1.52		
Parental work					
Yes	325 (91.3)	25.5	1	1.04-2.59	0.032*
No	31 (8.7)	41.9	1.64		
Maternal work					
No	256 (70.5)	28.1	1	0.56-1.23	0.358
Yes	107 (29.5)	23.4	0.83		
Number of residents in the household					
≤5	273 (74.6)	23.4	1	1.11-2.20	0.011*
>5	93 (25.4)	36.6	1.56		
Sewerage system					
Yes	209 (57.1)	24.4	1	0.87-1.72	0.236
No	157 (42.9)	29.9	1.23		
Sanitary installation					
Yes	362 (98.9)	26.7	1	0.25-6.25	0.788
No	4 (1.1)	33.3	1.25		
Garbage collection					
Yes	362 (98.9)	26.8	1	0.17-5.15	0.937
No	4 (1.1)	25.0	0.93		
Filtered water					
Yes	336 (91.8)	25.0	1	1.22-2.86	0.004*
No	30 (8.2)	46.7	1.87		

PR: prevalence ratio; 95%CI: 95% confidence intervals; ^a^Pearson’s chi-squared test; *statistical significance.

It was observed that 7.7% of the children were never breastfed and 15.2% never received exclusive breastfeeding, with the median breastfeeding time being 270 days. Most mothers reported weekly consumption of meats and/or viscera by children, however, 39.4% reported that children did not consume beans daily. It was also found that more than half of the children (58.9%) never took any iron supplements (Table 2).

**Table 2:** Crude prevalence rates of anemia and its 95% confidence intervals, according to the characteristics of mothers, breastfeeding practices and consumption of iron sources of children aged 6 to 23 months receiving care at Health Units of Vitória da Conquista, Bahia, Brazil, 2010/2011 (n=366).

	n (%)	Prevalence of anemia (%)	PR (crude)	95%CI	p-value^a^
Maternal age (years)					
≥20	314 (86.5)	26.4	1	0.73-1.84	0.532
<20	49 (13.5)	30.6	1.16		
Number of children					
≤3	320 (87.4)	23.1	1	1.60-3.18	<0.001*
>3	46 (12.6)	52.2	2.26		
Number of children aged <5 years					
<2	284 (77.6)	23.6	1	1.13-2.27	0.008*
≥2	82 (22.4)	37.8	1.60		
Type of delivery					
Normal	146 (40)	24.0	1	0.84-1.71	0.316
Cesarean	219 (60)	28.8	1.20		
Breastfeeding					
Yes	337 (92.3)	27.6	1	0.29-1.46	0.295
No	28 (7.7)	17.9	0.65		
Exclusive breastfeeding					
Yes	307 (84.8)	28.0	1	0.41-1.25	0.237
No	55 (15.2)	20.0	0.71		
Time of exclusive breastfeeding					
6 months	154 (50.5)	22.14	1	1.17-2.08	0.279
<6 months />6 months	151 (49.5)	31.40	1.42		
Consumption of meat and/or viscera					
≥once a week	306 (84.5)	23.2	1	1.41-2.83	<0.001*
<once a week	56 (15.5)	46.4	2.00		
Bean consumption					
Daily	214 (60.6)	21.5	1	1.14-2.27	0.007*
Non daily	139 (39.4)	34.5	1.61		
Consumption of dark green leafy vegetables					
Daily	48 (13.3)	20.8	1	0.74-2.38	0.336
Non daily	314 (86.7)	27.7	1.33		
Use of iron supplement					
Yes	148 (41.1)	18.2	1	1.22-2.68	0.003*
No	212 (58.9)	33.0	1.81		

PR: prevalence ratio; 95%CI: 95% confidence intervals; ^a^Pearson’s chi-squared test; *statistical significance.

The frequency of low birth weight and prematurity was 10.2 and 7.1%, respectively. In the evaluation of nutritional status, 4.4% of the children presented low weight for age, 5.6% underweight and 13.6% short stature (Table 3).

**Table 3:** Crude prevalence rates of anemia and its 95% confidence intervals, according to the characteristics of the children aged 6 to 23 months receiving care at Health Units of Vitória da Conquista, Bahia, Brazil, 2010/2011 (n=366).

	n (%)	Prevalence of anemia (%)	PR (crude)	95%CI	p-value^a^
Gender					
Female	183 (50.0)	27.9	1	0.66-1.29	0.637
Male	183 (50.0)	25.7	0.92		
Age (months)					
12 to 23	199 (54.4)	18.6	1	1.38-2.80	<0.001*
6 to 11	167 (45.6)	36.5	1.96		
Skin color					
White	188 (51.2)	25.0	1	0.79-1.58	0.518
Non white	175 (48.2)	28.0	1.12		
Gestational age at birth (weeks)					
≥37	338 (92.9)	26.9	1	0.42-1.77	0.677
<37	26 (7.1)	23.1	0.86		
Weight at birth (g)					
≥2500	326 (89.8)	26.4	1	0.66-1.91	0.657
<2500	37 (10.2)	29.7	1.13		
Previous hospitalization					
No	249 (68.2)	26.9	1	0.69-1.43	0.971
Yes	116 (31.8)	26.7	0.99		
Weight/age index (score Z)					
≥-2	344 (95.6)	27.0	1	0.39-2.20	0.860
<-2	16 (4.4)	25.0	0.92		
Height/age index (score Z)					
≥-2	311 (86.4)	27.0	1	0.60-1.62	0.944
<-2	49 (13.6)	26.5	0.98		
Weight/height ratio (score Z)					
≥-2	340 (94.4)	27.1	1	0.42-2.02	0.842
<-2	20 (5.6)	25.0	0.92		

PR: prevalence ratio; 95%CI: 95% confidence intervals; ^a^Pearson’s chi-squared test; *statistical significance.

In the bivariate analysis, among the variables of Block 1, family income, paternal schooling, maternal schooling, paternal work, number of residents in the household and the presence of filtered water were associated with anemia (p<0.05). There were higher prevalences of the outcome among children: with a family income equal to or lower than one minimum wage, with paternal and maternal schooling being less than eight years of study, with parents who did not work, who lived in households with more than five residents, and who did not have filtered water (Table 1).

Among the variables in Block 2, number of children, number of children aged less than five years, meat and/or viscera consumption, bean consumption and use of iron supplements were also significantly associated with anemia (p<0.05). Higher prevalences of anemia were observed in children of mothers with more than three children, with two or more children aged under five years, as well as in children who consumed meat and/or viscera less than once a week, who did not ingest beans daily, and who never used iron supplements (Table 2).

Regarding the individual characteristics (Block 3), only the age variable was included in the multiple regression model, which was significantly associated with anemia, with a higher prevalence of outcome in children aged 6 to 11 months (Table 3).

Considering a p<0.20 in the bivariate analysis, the variables of each block above were then inserted into the multivariate model and adjustments were made according to the hierarchical conceptual model. In the hierarchical multivariate analysis, it was observed that some variables significantly associated with anemia in the bivariate analysis lost significance and were not maintained in the models (Table 4).

**Table 4:** Multivariate analysis using Poisson regression for anemia and associated factors in children aged 6 to 23 months receiving care at Health Units of Vitória da Conquista, Bahia, Brazil, 2010/2011 (n=366).

	Model 1		Model 2		Model 3	
	PR	95%CI	PR	95%CI	PR	95%CI
Block 1						
Family income (minimum age)						
>1	1	1.03-2.18*	1	0.93-2.07	1	1.01-2.20*
≤1	1.50		1.39		1.49	
Number of residentes in the household						
≤5	1	1.07-2.11*	1	0.70-1.68	1	0.70-1.60
>5	1.50		1.08		1.06	
Filtered water						
Yes	1	1.11-2.56*	1	0.90-2.23	1	0.91-2.30
No	1.68		1.41		1.45	
Block 2						
Number of children						
≤3			1	1.01-2.68*	1	1.02-2.57*
>3			1.64		1.62	
Consumption of meat and/or viscera						
≥once a week			1	1.24-2.58**	1	0.97-2.07
< once a week			1.78		1.42	
Block 3						
Age (months)						
12 to 23					1	1.20-2.55**
6 to 11					1.75	
Akaike Criterion	432.07		422.77		418.67	

Model 1: adjusted between the variables in the block on structural processes of the society; Model 2: adjusted between the variables in the blocks on structural processes of the society and structural processes of the child’s immediate environment; Model 3: adjusted between the variables in the blocks on structural processes of the society, structural processes of the child’s immediate environment and individual processes of the child; PR: prevalence ratio; 95%CI: 95% confidence intervals; *p<0.05; **p<0.01.

In the distal block (Block 1), anemia association was observed with variables family income, number of residents in the household and filtered water. The prevalence of anemia was 50% higher among children with a family income equal to or lower than one minimum wage and living in households with more than five residents, compared to those with a family income higher than one minimum wage and living in households with five residents or less, respectively. In addition, the prevalence of anemia was 68% higher among children who did not have access to filtered water when compared to those who had (Table 4 - Model 1).

In Block 2, after adjustment for the variables of the same block and Block 1, only the variables number of children and consumption of meat and/or viscera remained associated with the outcome. The prevalence of anemia was 64% higher in the children of mothers with more than three children, in relation to those whose mothers had up to three children, and 78% higher in children who consumed meat and/or viscera less than once a week, when compared to those consumed it at least once a week. (Table 4 - Model 2).

In the more proximal block (Block 3), the age variable remained associated with anemia, and its prevalence was 75% higher among children aged 6 to 11 months, when compared to children aged 12 to 23 months (Table 4 - Model 3). For all models, the AIC estimate decreased with the adjustment of the variable blocks.

## DISCUSSION

According to the WHO,^6^ the prevalence of anemia in children evaluated in this study (26.8%) is characterized as a moderate public health problem, and is even more pronounced in the 6 to 11 months age group (36.5%). A similar result was observed in the study by Silveira et al.,^16^ in which a prevalence of 28.8% of anemia was observed in the children evaluated. However, other surveys conducted in the country observed higher prevalences.^4^ ^,^ ^5^ This is the case of a systematic review with a meta-analysis of the prevalence of anemia in Brazilian children, according to different epidemiological scenarios, which concluded that anemia reaches levels higher than 40% in children in the country, and in children receiving care in health services, prevalence ranged from 55.1 to 89.1%, with a weighted average of 60.2%.^3^ Public policies aimed at reducing the prevalence of anemia have been implemented in Brazil; however, difficulties are still observed in the prevention and control of iron deficiency, especially in children.^10^

Regarding socioeconomic characteristics, studies have referred to income as an important determinant of anemia.^4^ ^,^ ^5^ The social and economic conditions of the lower income classes favor the development of anemia, either due to a quantitatively and qualitatively inadequate diet, or due to the precariousness of environmental sanitation or other indicators that could directly or indirectly contribute to its high prevalence.^17^

The present study observed that children living in households with more than five people had a higher prevalence of anemia, similar to that found in other studies.^5^ ^,^ ^18^. According to Leal et al.*,* ^19^ the greater risk of anemia in children with unfavorable housing conditions, such as a large number of residents, could be explained by the reduction in the economic accessibility of these families, leading to a per capita reduction of food and , consequently, to the reduction in the intake of iron-rich foods. In addition, Neuman et al.^5^ discuss the possibility of some factor related to the increase of infections - more frequent with family agglomeration -, although, as in this study, the hospital admission variable was not significant.

The association between the use of unfiltered water and the higher prevalence of anemia observed in this study could be related to the fact that this could be an important vehicle for infection by intestinal parasites.^20^ Spoiling enteroparasites are an important factor in the etiology of dietary anemia and caloric protein malnutrition, since adequate nutritional status depends not only on food intake, but also on its efficient biological utilization, which may be compromised in cases of infestation by this type of enteroparasitas.^21^

In the bivariate analysis, socioeconomic variables (maternal and paternal education, and paternal work) had a significant association with anemia. However, when analyzed in conjunction with the other socioeconomic variables, they lost their significance and, therefore, did not remain in the multivariate analysis. Maternal and paternal education had been associated with anemia, since the greater knowledge of diseases has repercussions on preventive care and the search of health services; in addition, a better schooling level favors the insertion of the individual in the labor market and the increase of income.^22^

Regarding maternal variables, the number of children has also been related to the development of anemia in other studies.^4^ ^,^ ^19^ In a family with a large number of children, there is an increased demand for food, which is not always available in terms of quality and quantity for all members, as well as the reduction of health and food care provided to children.^17^

Regarding dietary practices, they play a fundamental role in the development and prevention of iron-deficiency anemia. In this study, an association between low frequency of consumption of meat and/or viscera and a higher prevalence of anemia was observed. According to the recommendation of the Ministry of Health,^23^ sources of animal protein, such as meat and viscera, have high bioavailability iron, so that, from six months on, these foods must be present at least once a week in savory baby foods offered to the children, ensuring an adequate supply of this micronutrient, preventing anemia.

In the bivariate analysis, it was observed that children who did not consume beans daily and who did not use an iron supplement were more likely to have anemia. However, when associated with other maternal variables, breastfeeding practices and consumption of iron-rich foods, these variables lost statistical significance. It is known that prophylactic iron supplementation is a greatly important strategy for the prevention of anemia, and is recommended by the Ministry of Health.^24^ In contrast to the present study, a survey conducted in Viçosa, Minas Gerais, with infants, found a greater chance of anemia (*Odds Ratio* - OR 2.39, 95% confidence interval -95%CI 1.17-4.90) in children who did not consume iron supplementation when compared to those who consumed it. Regarding the intake of this supplement, consumption by the infants in Viçosa was even lower (21.5%) than in Vitória da Conquista (41.1%).^25^ This study did not aim to know the factors associated with the low consumption of iron supplementation; however, it is assumed that it may be associated to the inadequate distribution of the medication in the municipality, to the absence of prescription by health care professionals of basic care, or to parents or guardians not administering the supplement. Thus, more studies are needed to know the factors associated with the low consumption of prophylactic iron by infants in the municipality.

In relation to age, there was a higher prevalence of the outcome in children aged 6 to 11 months, and this younger age group has also been reported in the literature as being at greater risk for anemia,^26^ ^,^ ^27^ due to the child’s accelerated growth and development, leading to increased iron requirements in this period.^28^

The design of the present study is transversal, whose limitation is the establishment of a temporal relation between some variables of exposure with the outcome. However, this study discusses the importance of appropriate data analysis strategies to evaluate the determinants of health conditions. The present study used the hierarchical conceptual model to conduct the multivariate analysis, considering the hierarchical relationships among the variables, which allows the interpretation of the results in light of social and biological knowledge.^29^

As a limitation, the diagnosis of anemia was made by Hemocue, which evaluates only hemoglobin levels and may result in false negative diagnosis. However, the use of this method is validated for field research and has been widely used in epidemiological research; in addition, it has sufficient specificity and sensitivity to detect altered levels of hemoglobin.^30^

The results of the present study show that anemia in infants assisted in Health Units of the urban area of the municipality of Vitória da Conquista is a moderate public health problem, mainly in children aged 6 to 11 months, besides being associated with socioeconomic, maternal and dietary factors. It is emphasized that studies on anemia and its determinants are of great relevance, as their results can guide the implementation of measures aimed at reducing and preventing this nutritional deficiency. In this context, it is extremely important to permanently monitor the strategies used to control this disease so as to ensure their effectiveness.
